# Baseline Graded Prognostic Assessment Is Associated with Postoperative Health-Related Quality of Life Recovery After Brain Metastasis Resection: A Prospective Cohort Study

**DOI:** 10.3390/jcm15166166

**Published:** 2026-08-08

**Authors:** Kadir Cetinkaya, Yasar Ünsal, Mert Yığıt, Erva Eser, Mehmet Özgür Özates, Atilla Kazancı

**Affiliations:** 1 Department of Neurosurgery, Hıtıt University Corum Erol Olcok Training and Research Hospital, Corum 19000, Türkiye; 2 Department of Neurosurgery, Bilkent City Hospital, Ankara 06800, Türkiye; yaasar40@gmail.com (Y.Ü.); dr.yigitmert@gmail.com (M.Y.); 3 Department of Biostatistics, Faculty of Veterinary Medicine, Kırıkkale University, Kırıkkale 71450, Türkiye; ervaeser@kku.edu.tr; 4 Department of Neurosurgery, Yıldırım Beyazıt University, Ankara 06800, Türkiye

**Keywords:** brain metastasis, neurosurgery, health-related quality of life, EQ-5D-5L, graded prognostic assessment

## Abstract

**Objective:** The objective was to evaluate longitudinal changes in health-related quality of life (HRQoL) following surgical resection of brain metastases and investigate the prognostic value of the Graded Prognostic Assessment (GPA) score in predicting HRQoL improvement. **Methods:** This prospective observational study included 42 consecutive adult patients undergoing microsurgical resection for histopathologically confirmed brain metastases. HRQoL was assessed using the EuroQol Five-Dimension Five-Level questionnaire (EQ-5D-5L) questionnaire and EuroQol Visual Analogue Scale (EQ-VAS) preoperatively and at 1 and 6 months postoperatively. Patients were stratified according to their preoperative GPA scores, and longitudinal changes in HRQoL were analyzed using non-parametric statistical methods. **Results**: Mean EQ-VAS scores exhibited a significant postoperative increase from 38.3 ± 19.3 at baseline to 77.6 ± 19.3 at one month and 75.7 ± 16.7 at six months (*p* < 0.001). Significant improvements were observed across all five EQ-5D-5L dimensions (all *p* < 0.001). Patients with favorable GPA scores (3.0–4.0) demonstrated greater improvements in EQ-VAS than those in lower GPA categories at one month (*p* = 0.035) and six months (*p* = 0.013). Higher baseline GPA scores were positively correlated with HRQoL improvement at both follow-up time points (ρ = 0.51 and ρ = 0.50, respectively; both *p* < 0.001). Changes in clinician-rated Karnofsky Performance Status (KPS) did not differ significantly across GPA categories (*p* = 0.731). **Conclusions:** Surgical resection of brain metastases significantly improves patient-reported HRQoL. The GPA score is a significant predictor of postoperative HRQoL gain, whereas traditional clinician-rated scales like KPS may lack the sensitivity to capture these patient-centered improvements. However, the findings regarding GPA subgroups should be considered exploratory due to the limited number of patients in certain tiers.

## 1. Introduction

Brain metastases (BM) are the most common intracranial tumors in adults, occurring in approximately 20–40% of patients with systemic cancer and representing a major cause of neurological morbidity and impaired quality of life [1]. Beyond their adverse impact on survival, BM frequently result in motor deficits, cognitive dysfunction, seizures, and elevated intracranial pressure, all of which substantially compromise health-related quality of life (HRQoL) [2]. Consequently, contemporary management strategies increasingly emphasize not only prolonging survival but also preserving functional independence and patient-reported well-being [3].

Surgical resection remains a cornerstone of treatment for selected patients with brain metastases, particularly those with large symptomatic lesions causing mass effect, obstructive hydrocephalus, or requiring histopathological confirmation [4,5]. In appropriately selected patients, surgery provides rapid neurological improvement, facilitates subsequent oncological treatment, and contributes to durable local disease control. Despite these well-recognized clinical benefits, postoperative outcomes continue to be evaluated predominantly using clinician-reported measures, while patient-perceived recovery has received comparatively less attention [6].

Recent advancements in the systemic therapeutic landscape, including targeted molecular therapies and immune checkpoint inhibitors, have fundamentally altered the natural history of brain metastases, leading to significantly prolonged survival even in patients with advanced disease [7]. This shift has catalyzed a paradigmatic transition in neuro-oncology, moving beyond binary outcomes of survival versus mortality toward the concept of ‘precision survivorship’ [8]. In this context, the preservation of functional integrity and the mitigation of neurocognitive decline have emerged as primary therapeutic objectives [9,10]. However, the longitudinal trajectory of recovery following surgical intervention remains highly heterogeneous, influenced by a complex interplay of intracranial tumor dynamics, systemic disease burden, and the cumulative toxicity of multimodal treatments [6,10]. Despite the increasing emphasis on patient-centered care, there remains a critical evidence gap in identifying robust clinical markers that can reliably predict the magnitude of quality-of-life gains, necessitating a more granular exploration of established prognostic frameworks like the Graded Prognostic Assessment (GPA) in the context of functional recovery.

Functional recovery after brain metastasis surgery has traditionally been assessed using clinician-rated instruments such as the Karnofsky Performance Status (KPS). Although KPS remains an established indicator of functional capacity, it may not adequately reflect patients’ subjective perceptions of health status and daily functioning [11,12]. In contrast, patient-reported outcome measures (PROMs), including the EuroQol Five-Dimension Five-Level questionnaire (EQ-5D-5L), provide a comprehensive assessment of health-related quality of life across physical, psychological, and social domains. Consequently, PROMs have become increasingly recognized as essential endpoints in neuro-oncology research and patient-centered clinical care [13,14].

The GPA is a widely validated prognostic model that estimates survival in patients with brain metastases using readily available clinical variables, including age, KPS, number of brain metastases, and extracranial disease status [15]. While GPA is routinely used to support treatment selection and survival estimation, its relationship with postoperative patient-reported quality of life remains largely unknown. Because the variables incorporated into GPA collectively reflect baseline disease burden and physiological reserve, they may also influence the extent of functional recovery perceived by patients after surgery [16,17].

Therefore, this prospective study aimed to evaluate longitudinal changes in HRQoL following surgical resection of brain metastases using the EQ-5D-5L and EuroQol Visual Analogue Scale (EQ-VAS) and to determine whether baseline GPA is associated with the magnitude of postoperative HRQoL improvement. We prespecified the hypothesis that patients with more favorable GPA scores would experience greater gains in postoperative quality of life. By examining the relationship between an established survival prognostic tool and patient-reported recovery, this study seeks to provide evidence that may improve prognostic counseling and support more patient-centered surgical decision-making.

## 2. Methods

### 2.1. Study Design and Patient Population

This prospective observational cohort study was conducted at a single tertiary academic neurosurgical center between June 2025 and June 2026. Following the enrollment of the final patient in January 2026, the 6-month follow-up period concluded in June 2026, and the database was closed for analysis on 15 June 2026. Database closure and statistical analyses were performed only after all included patients had completed the scheduled 6-month postoperative follow-up. The study protocol was approved by the local Institutional Review Board/Ethics Committee before patient enrollment (Approval No. TABED 2-25-1248/2025) and was conducted in accordance with the Declaration of Helsinki and its subsequent amendments. Written informed consent was obtained from all participants.

**Figure 1:**
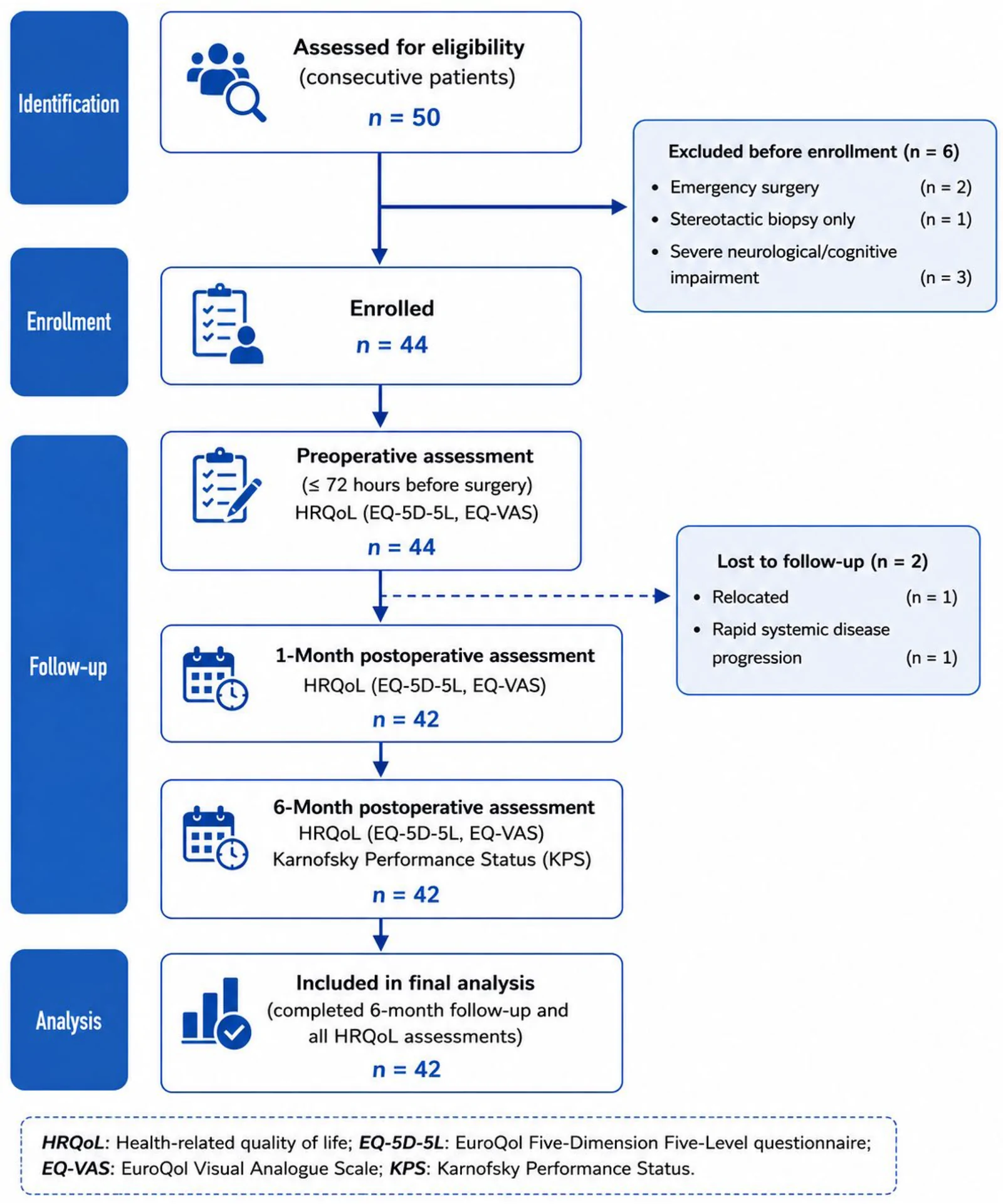
STROBE flow diagram illustrating patient screening, enrollment, follow-up, and final study cohort.

Fifty consecutive adult patients with radiologically suspected intracranial brain metastases who were scheduled for elective microsurgical resection were prospectively screened for eligibility. Six patients were excluded before enrollment, including two who underwent emergency surgery, one who underwent stereotactic biopsy without tumor resection, and three with severe neurological or cognitive impairment preventing reliable completion of the HRQoL questionnaires. During follow-up, two patients were lost before completing the scheduled postoperative assessments (one patient relocated and was unable to attend follow-up visits, and one patient experienced rapid systemic disease progression that precluded HRQoL assessment). Therefore, the final study cohort consisted of 42 patients who completed all evaluations, with no missing HRQoL data (Figure 1).

Because of the exploratory nature of this prospective observational study, no formal a priori sample size calculation was performed. The study population therefore comprised all consecutive eligible patients treated during the study period.

### 2.2. Eligibility Criteria

Patients were eligible if they were ≥18 years of age, had radiologically suspected and histopathologically confirmed brain metastasis originating from a solid primary malignancy, and underwent elective craniotomy with microsurgical tumor resection. Participants were also required to be cognitively able to understand and independently complete the HRQoL questionnaires. Patients undergoing emergency surgery, stereotactic biopsy without tumor resection, or those with severe neurological or cognitive impairment precluding reliable questionnaire completion were excluded.

### 2.3. Clinical Data Collection

Baseline demographic and clinical characteristics were prospectively recorded at study enrollment. Baseline demographic and clinical characteristics, including primary tumor origin and preoperative clinical presentations, were recorded at study enrollment and are presented in Table 1. Variables included age, sex, primary tumor type, preoperative KPS, number of intracranial metastatic lesions, presence of extracranial metastatic disease, and preoperative radiological findings. Functional status was assessed using the KPS before surgery and at each postoperative follow-up visit. To further characterize the study cohort, extent of resection (gross total vs. subtotal), tumor volume, and postoperative adjuvant therapies were also recorded. These variables were collected for descriptive purposes and were not included as primary explanatory variables in the present analysis. However, due to the modest sample size and the potential for model overfitting, these variables were used for descriptive purposes only and were not included as primary explanatory variables or covariates in the multivariable statistical models.

### 2.4. Prognostic Stratification

Patients were stratified according to the original GPA described by Sperduto et al. [18], the first widely adopted prognostic scoring system specifically developed for patients with brain metastases. GPA scores were calculated using four established clinical variables: age, preoperative KPS, number of intracranial metastatic lesions, and presence of extracranial metastatic disease. The total GPA score ranged from 0.0 to 4.0, with higher scores indicating a more favorable prognosis.

Patients were categorized as follows:

Poor prognosis: GPA 0.0–1.0;
Intermediate prognosis: GPA 1.5–2.5;
Favorable prognosis: GPA 3.0–4.0.

The original GPA was selected because it relies exclusively on universally available clinical variables, allowing consistent prognostic stratification across patients with different primary tumor types. Although originally developed to predict overall survival, this study explored whether baseline GPA was also associated with postoperative recovery in patient-reported health-related quality of life.

### 2.5. Health-Related Quality of Life Assessment

Health-related quality of life was prospectively assessed using the validated EQ-5D-5L together with the EQ-VAS. Assessments were performed at three predefined time points: within 72 h before surgery, at the first postoperative month, and at the sixth postoperative month. The EQ-5D-5L evaluates five dimensions of health status: mobility, self-care, usual activities, pain/discomfort, and anxiety/depression. Each dimension is scored on a five-level ordinal scale ranging from no problems to extreme problems. Overall self-perceived health was assessed using the EQ-VAS, in which patients rated their health on a scale from 0 (worst imaginable health) to 100 (best imaginable health). The primary outcome was postoperative change in global health status, expressed as the difference between postoperative and baseline EQ-VAS scores. Secondary outcomes included longitudinal changes in the individual EQ-5D-5L dimensions and postoperative changes in KPS.

### 2.6. Statistical Analysis

Statistical analyses were performed using R software (version 4.3.3.1; R Foundation for Statistical Computing, Vienna, Austria). Distributional assumptions were evaluated using graphical inspection and the Shapiro–Wilk test. Because of the modest sample size and the ordinal nature of the EQ-5D-5L descriptive dimensions, non-parametric statistical methods were applied throughout. Continuous variables are presented as median (interquartile range [IQR]) and additionally as mean ± standard deviation (SD) for descriptive comparison with previous clinical studies. Categorical variables are presented as frequencies and percentages. Baseline continuous variables were compared across GPA categories using the Kruskal–Wallis test, whereas categorical variables were analyzed using Pearson’s chi-square test or Fisher’s exact test, as appropriate. Longitudinal changes in EQ-VAS and EQ-5D-5L dimensions were assessed using the Friedman test. Preoperative and postoperative KPS values were compared using the Wilcoxon signed-rank test. Pairwise comparisons of EQ-VAS scores were performed using the Wilcoxon signed-rank test with Holm correction for multiple comparisons, and effect sizes were quantified using Kendall’s W. The five EQ-5D-5L domain-level tests were interpreted using Holm adjustment for multiple comparisons. Associations between continuous GPA scores and postoperative HRQoL improvement were evaluated using Spearman’s rank correlation coefficient. ε^2^ was calculated as the effect-size measure for Kruskal–Wallis comparisons. As sensitivity analyses, separate baseline-adjusted linear regression models (ANCOVA approach) were fitted for 1-month and 6-month EQ-VAS, with GPA entered as the primary predictor and baseline EQ-VAS included as a covariate. In addition, to confirm the robustness of our findings, we re-evaluated the association between continuous GPA and HRQoL improvement after excluding the poor-prognosis subgroup (*n* = 3). All statistical tests were two-sided, and a *p*-value < 0.05 was considered statistically significant. Because no missing HRQoL data were observed, no data imputation was performed. Given the exploratory design and the limited number of patients in the poor-prognosis GPA subgroup, subgroup analyses should be interpreted as exploratory and hypothesis-generating.

## 3. Results

### 3.1. Patient Characteristics

A total of 42 patients with histologically confirmed brain metastases who underwent surgical resection completed the study and were included in the final analysis. Table 1 was expanded to include primary tumor origin and preoperative clinical presentations. The most common primary malignancy was lung carcinoma (35.7%), followed by renal cell carcinoma (16.7%). The most frequent preoperative symptoms were headache (50.0%) and focal motor deficits (38.1%). According to the preoperative GPA, 3 patients (7.1%) were classified as having poor prognosis, 24 (57.1%) intermediate prognosis, and 15 (35.7%) favorable prognosis. Baseline demographic and clinical characteristics are presented in Table 1. The mean age of the study cohort was 61.7 ± 8.7 years, and the mean preoperative KPS was 77.9 ± 14.7. Baseline EQ-VAS scores were comparable across the three GPA groups (*p* = 0.615), with no significant between-group differences in age (*p* = 0.244) or preoperative KPS (*p* = 0.308). As defined by the study design, GPA scores differed significantly among the prognostic groups (*p* < 0.001). The prevalence of extracranial metastatic disease also varied significantly across GPA categories (*p* < 0.001), whereas the number of intracranial metastatic lesions was similar among groups (*p* = 0.442).

### 3.2. Longitudinal Changes in Global Health-Related Quality of Life

**Figure 2:**
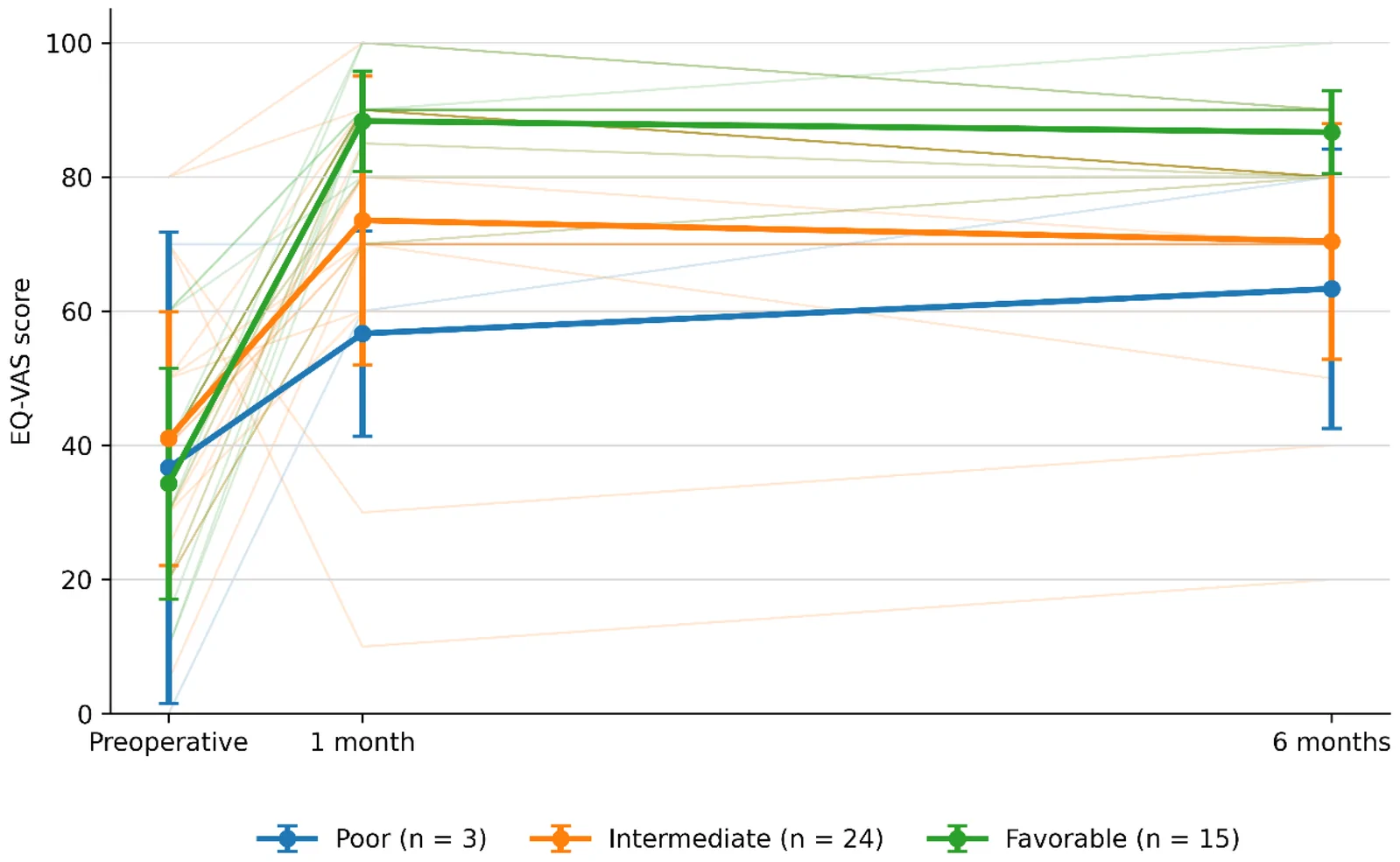
Longitudinal changes in patient-reported global health status (EQ-VAS) according to baseline GPA category. Mean EQ-VAS scores were assessed preoperatively and at 1 and 6 months after surgical resection. Patients were stratified into poor (GPA 0.0–1.0), intermediate (GPA 1.5–2.5), and favorable (GPA 3.0–4.0) prognostic groups. Points represent group means and error bars represent standard deviations. Because the poor-prognosis group was small, category-specific trajectories should be interpreted descriptively. EQ-VAS, EuroQol Visual Analogue Scale; GPA, Graded Prognostic Assessment.

Longitudinal changes in patient-reported global health status are illustrated in Figure 2. Mean EQ-VAS increased from 38.3 ± 19.3 preoperatively to 77.6 ± 19.3 at the first postoperative month and remained stable at 75.7 ± 16.7 at six months.

Repeated measures analysis demonstrated a significant overall change in EQ-VAS across the three assessment time points (Friedman test, *p* < 0.001). Compared with baseline, EQ-VAS was higher at the first postoperative month (mean change, 39.3 ± 29.3 points; Holm-adjusted *p* < 0.001) and at the sixth postoperative month (mean change, 37.4 ± 27.2 points; Holm-adjusted *p* < 0.001). EQ-VAS did not differ between the first- and sixth-month assessments (Holm-adjusted *p* = 0.275). These within-cohort postoperative changes should not be interpreted as evidence of a causal treatment effect.

### 3.3. Association Between GPA and Postoperative HRQoL Recovery

**Figure 3:**
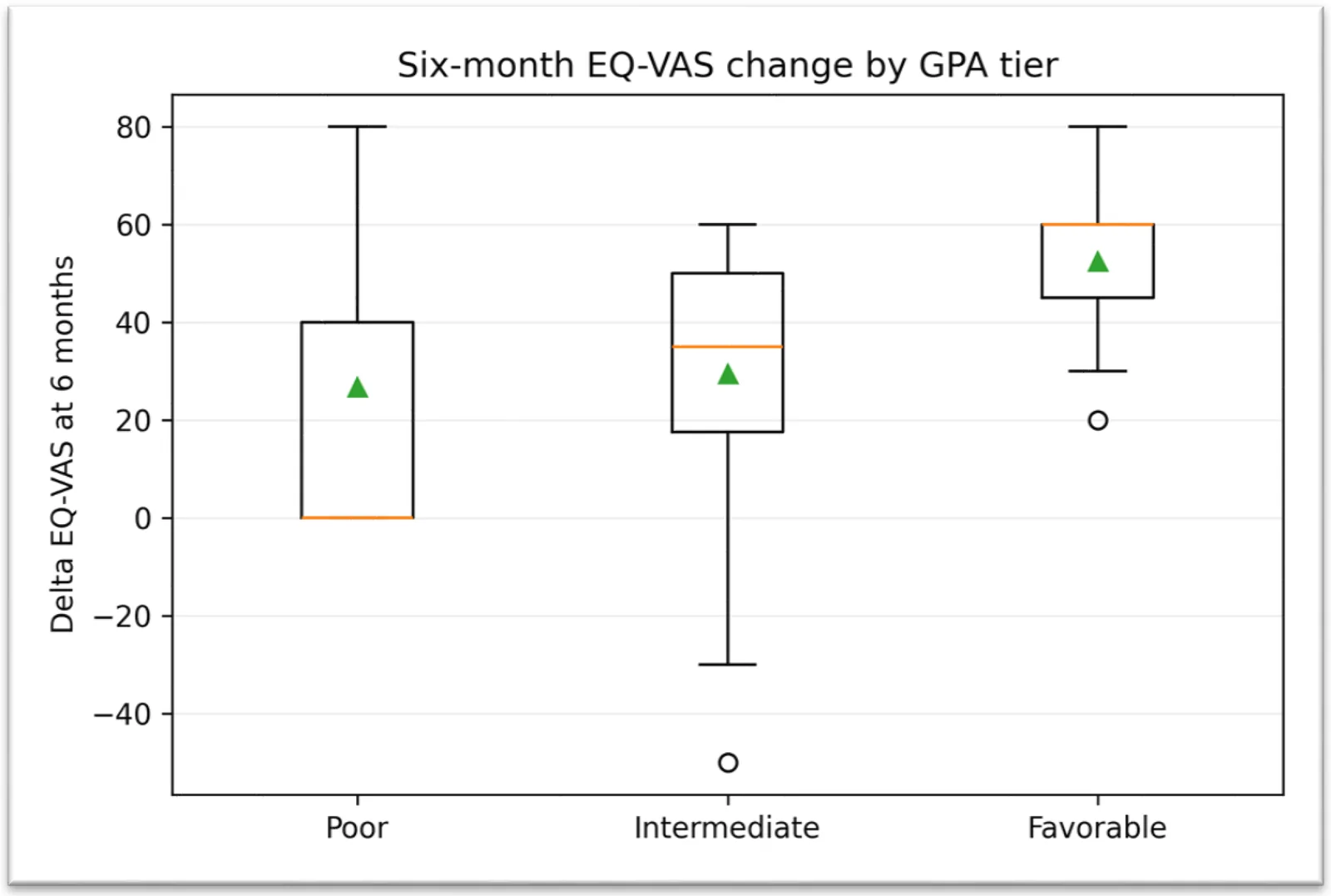
Six-month change in patient-reported global health status according to baseline GPA category. The figure illustrates the change in EQ-VAS score from baseline to the sixth postoperative month (EQ-VAS) across baseline GPA categories. Positive EQ-VAS values indicate greater improvement in patient-reported global health status between baseline and the sixth postoperative month. GPA categories were defined as poor (0.0–1.0, *n* = 3), intermediate (1.5–2.5, *n* = 24), and favorable (3.0–4.0, *n* = 15). The triangle denotes the mean, the box denotes the interquartile range, and whiskers indicate the non-outlying range. Circles indicate outlier values. Given the small poor-prognosis subgroup, the figure is descriptive. EQ-VAS, EuroQol Visual Analogue Scale; GPA, Graded Prognostic Assessment.

Postoperative EQ-VAS change according to baseline GPA is presented in Table 2 and Figure 3. Because the poor-prognosis subgroup included only three patients, category-based comparisons were considered exploratory. Continuous GPA analyses were therefore emphasized.

In exploratory comparisons across GPA categories, patients in the favorable GPA group demonstrated the greatest increase in EQ-VAS at the first postoperative month (Kruskal–Wallis, *p* = 0.035, ε^2^ = 0.12) and at the sixth postoperative month (*p* = 0.013, ε^2^ = 0.17). When GPA was analyzed as a continuous variable, higher baseline GPA scores were associated with greater postoperative HRQoL improvement. Significant positive correlations were observed between GPA and ΔEQ-VAS at the first postoperative month (Spearman’s ρ = 0.51, *p* < 0.001) and at the sixth postoperative month (ρ = 0.50, *p* < 0.001).

As sensitivity analyses adjusting for baseline EQ-VAS, separate linear regression models showed that each one-point higher recorded GPA was associated with a 11.1-point higher EQ-VAS at 1 month (95% CI, 5.0–17.1; *p* = 0.001) and a 9.7-point higher EQ-VAS at 6 months (95% CI, 4.4–14.9; *p* = 0.001). In an additional sensitivity analysis excluding the poor-prognosis subgroup, the association between continuous GPA and EQ-VAS improvement remained significant at both 1 month (Spearman’s ρ = 0.50, *p* = 0.001) and 6 months (ρ = 0.55, *p* < 0.001; *n* = 39).

Postoperative changes in clinician-rated functional status were not associated with GPA, either when analyzed by prognostic category (ΔKPS, *p* = 0.731) or as a continuous score (ρ = 0.01, *p* = 0.933). Because only three patients were included in the poor-prognosis subgroup, comparisons across GPA categories should be interpreted as exploratory.

### 3.4. Changes in EQ-5D-5L Dimensions

Longitudinal changes in the five EQ-5D-5L descriptive dimensions are summarized in Table 3. Significant changes were observed across all domains during follow-up (*p* < 0.001), with moderate-to-large effect sizes (Kendall’s W ranging from 0.43 to 0.59). All five domain-level findings remained statistically significant after Holm adjustment for multiple comparisons.

Median scores decreased substantially during follow-up, indicating fewer limitations in mobility, self-care, usual activities, pain/discomfort, and anxiety/depression. These changes were evident at the first postoperative month and were maintained at six months. To facilitate clinical interpretation, the proportion of patients reporting favorable health states (EQ-5D levels 1–2) was also evaluated. The percentage of patients with favorable responses increased consistently across all five dimensions throughout follow-up. At six months, favorable mobility increased from 31.0% to 92.9%, self-care from 21.4% to 97.6%, and usual activities from 16.7% to 90.5%. Favorable responses for pain/discomfort increased from 14.3% preoperatively to 100% at six months, while favorable anxiety/depression scores increased from 16.7% to 76.2% (Supplementary Table S1).

### 3.5. Functional Outcomes

Changes in clinician-rated functional performance were evaluated using the KPS. In the overall cohort, mean KPS was 77.9 ± 14.7 preoperatively and 80.0 ± 11.7 postoperatively. The paired change was not statistically significant (Wilcoxon signed-rank test, W = 153.0, *p* = 0.372).

Postoperative change in KPS was not associated with either baseline GPA category (Kruskal–Wallis *p* = 0.731) or continuous GPA score (Spearman’s ρ = 0.01, *p* = 0.933). Because most patients had relatively preserved baseline functional status, these findings should not be interpreted as evidence that KPS is less responsive than patient-reported outcome measures.; Supplementary Table S2).

## 4. Discussion

Our prospective observational cohort study demonstrates that surgical resection of brain metastases is associated with substantial and sustained improvements in patient-reported HRQoL. Global health status, measured by the EQ-VAS, improved markedly during the first postoperative month and remained stable at six months (Table 2), indicating that most recovery occurred early after surgery and was maintained throughout follow-up. These findings, while descriptive, reinforce the role of surgery as a potent tool for symptomatic relief and functional restoration in a population burdened by significant neurological morbidity [19,20]. However, given the uncontrolled observational design, these improvements should be interpreted as associations within the postoperative period rather than definitive causal effects of surgery alone.

The benefits of surgery extended beyond global health perception. Significant improvements were observed across all five EQ-5D-5L domains, including mobility, self-care, usual activities, pain/discomfort, and anxiety/depression (Table 3). The greatest improvements occurred in physical functioning and pain/discomfort, with a marked increase in the proportion of patients reporting favorable health states (EQ-5D levels 1–2). Collectively, these findings suggest that surgical resection alleviates both the neurological and psychological burden of metastatic brain disease, resulting in broad improvements in patients’ daily functioning and overall well-being, consistent with previous HRQoL studies in this population [20]. A notable observation in our longitudinal data was the apparent plateau in EQ-VAS scores between the one-month and six-month assessments. While this stability suggests that the primary gains from surgical intervention are realized early and maintained over time, it must be interpreted with caution. This trend may reflect a physiological stabilization of recovery; however, we cannot exclude the possibility of a “ceiling effect” inherent to the EQ-VAS instrument or a lack of sensitivity in the EQ-5D-5L to capture more nuanced, long-term improvements in this specific oncological cohort.

The principal finding of our study is the association between baseline GPA and postoperative HRQoL recovery (Figure 3). Although GPA was originally developed as a survival prognostic model, patients with more favorable baseline GPA scores demonstrated significantly greater improvements in EQ-VAS at both postoperative assessments. These findings suggest that the clinical parameters incorporated into GPA may reflect not only expected survival but also the potential for postoperative recovery from the patient’s perspective. The prognostic value of GPA for survival has been well established [21]; however, our results indicate that this routinely calculated score may provide additional clinically relevant information regarding quality-of-life trajectories after surgery [22]. The predictive capacity of GPA for HRQoL recovery may be attributed to its composite structure, integrating age, functional status, and systemic disease burden as indicators of overall physiological reserve. Patients with higher GPA scores may have greater resilience to the physiological stress of neurosurgical intervention, facilitating more favorable recovery trajectories. From a clinical standpoint, these findings highlight the potential role of GPA in preoperative counseling and shared decision-making by allowing clinicians to provide more individualized expectations regarding postoperative recovery. Nevertheless, these observations should be interpreted cautiously and require validation in larger prospective multicenter cohorts before routine application as a HRQoL prediction tool.

Another important observation was the discrepancy between patient-reported and clinician-rated outcomes. Despite marked improvements in EQ-VAS and all EQ-5D-5L dimensions, changes in KPS remained modest and were not associated with GPA. This finding is consistent with previous reports suggesting that clinician-rated performance scales may underestimate clinically meaningful improvements perceived by patients during recovery [23]. However, this discrepancy should not be interpreted as evidence of the superiority of one assessment over the other; rather, KPS and PROMs capture complementary dimensions of postoperative recovery. The limited change in KPS observed in our cohort may, in part, reflect a ceiling effect, as 78.6% of patients had a baseline KPS ≥70. In contrast, PROMs are more sensitive to subjective improvements in physical, emotional, psychological, and social well-being that may be insufficiently captured by traditional clinician-rated performance scales [24,25,26]. Together, these findings support the complementary use of PROMs alongside conventional functional assessments to provide a more comprehensive evaluation of surgical outcomes and to better reflect the patient’s perspective during postoperative recovery.

Looking forward, the evolution of prognostic modeling in neuro-oncology must transcend traditional clinical variables to incorporate the burgeoning field of molecular diagnostics [27]. The integration of primary tumor molecular profiles—such as EGFR mutations in lung carcinoma or BRAF status in melanoma—into patient-reported outcome models represents a promising avenue for future research [28,29]. Furthermore, the application of machine learning algorithms to large-scale, longitudinal HRQoL datasets could facilitate the development of dynamic, real-time predictive tools that account for the cumulative effects of adjuvant therapies and systemic disease progression [30,31]. By transitioning from static prognostic scores to multidimensional, molecularly integrated frameworks, clinicians can move closer to the goal of truly personalized surgical management, where interventions are tailored not only to survival probability but to the specific functional and subjective recovery potential of the individual patient.

Collectively, our findings advocate for a more holistic, patient-centered approach to evaluating surgical outcomes in neuro-oncology. Integrating GPA-based prognosticating with comprehensive HRQoL assessments allows for a more nuanced understanding of the surgical impact, ultimately facilitating more informed and individualized clinical management of patients with brain metastases.

## 5. Limitations

This study has several limitations that should be acknowledged. First, the relatively small sample size and single-center design may limit the generalizability of our findings. Second, the poor-prognosis GPA subgroup included only three patients, requiring cautious interpretation of subgroup analyses, which should be regarded as exploratory. Third, the use of a complete-case analysis introduces a survivorship bias, as patients who deteriorated rapidly or died before the 6-month assessment were not included in the final longitudinal analysis. This may lead to an overestimation of the average HRQoL benefit in the broader brain metastasis population. Furthermore, we were unable to include extent of resection, tumor volume, and adjuvant therapies as covariates in our analysis due to data limitations. These factors are known to influence HRQoL and could act as confounders in the GPA–HRQoL relationship. While primary tumor origin and clinical presentations are now reported descriptively in Table 1, the modest sample size precluded their inclusion in multivariable models. Consequently, the GPA–HRQoL relationship should be viewed as an exploratory association rather than an independent predictive effect. Additionally, the study did not calculate the EQ-5D-5L utility index because a validated country-specific value set for the Turkish population is currently unavailable. Although utility values can be estimated using value sets derived from other populations, this approach may introduce uncertainty due to differences in health state preferences. Consequently, we focused on longitudinal changes in EQ-5D-5L descriptive dimensions and EQ-VAS, which limits the interpretation of our findings in utility-based health economic evaluations. The absence of a longitudinal control group precludes direct comparison with nonsurgical treatment strategies. Finally, postoperative HRQoL may have been influenced by heterogeneous adjuvant oncological therapies that were not evaluated separately. Larger prospective multicenter studies with standardized treatment protocols are needed to validate these findings and further define the relationship between prognostic status and patient-reported recovery.

## 6. Conclusions

Surgical resection of brain metastases was associated with significant and sustained postoperative improvements in patient-reported health-related quality of life. Patients with more favorable baseline GPA scores experienced greater postoperative HRQoL gains, suggesting that GPA may provide clinically useful information beyond survival estimation. However, because of the uncontrolled prospective observational design, these findings should be interpreted as postoperative associations rather than evidence of a causal effect of surgery alone, as concomitant treatments and the natural postoperative recovery process may also have contributed to the observed improvements. If validated in larger prospective multicenter studies with appropriate comparator groups, GPA could complement existing prognostic assessment by helping clinicians anticipate postoperative quality-of-life recovery and facilitate more informed, patient-centered surgical decision-making.

## Figures and Tables

**Table 1:** Baseline demographic and clinical characteristics according to preoperative GPA category.

Characteristic	Overall (*n* = 42)	Poor (*n* = 3)	Intermediate (*n* = 24)	Favorable (*n* = 15)	*p*-Value
**Continuous variables**					
Age, years	61.7 ± 8.7	60.3 ± 11.7	62.9 ± 9.6	60.0 ± 6.9	0.244
Preoperative KPS	77.9 ± 14.7	66.7 ± 15.3	77.1 ± 15.5	81.3 ± 13.0	0.308
Baseline EQ-VAS	38.3 ± 19.3	36.7 ± 35.1	41.0 ± 18.9	34.3 ± 17.2	0.615
**Categorical variables**					
**Extracranial metastasis, *n* (%)**					
Present	16 (38.1)	3 (100.0)	13 (54.2)	0 (0.0)	**<0.001**
Absent	26 (61.9)	0 (0.0)	11 (45.8)	15 (100.0)	
**Number of brain metastases, *n* (%)**					
1	30 (71.4)	1 (33.3)	17 (70.8)	12 (80.0)	0.299
2–3	9 (21.5)	1 (33.3)	5 (20.8)	3 (20.0)	
>3	3 (7.1)	1 (33.3)	2 (8.4)	0 (0.0)	
**Primary tumor origin, *n* (%)**					
Lung carcinoma	15 (35.7)	—	—	—	—
Renal cell carcinoma	7 (16.7)	—	—	—	—
Ovarian carcinoma	4 (9.5)	—	—	—	—
Gastric carcinoma	4 (9.5)	—	—	—	—
Thyroid carcinoma	3 (7.1)	—	—	—	—
Melanoma	3 (7.1)	—	—	—	—
Other	3 (7.1)	—	—	—	—
**Preoperative clinical presentation †, *n* (%)**					
Headache	21 (50.0)	—	—	—	—
Focal motor deficit	16 (38.1)	—	—	—	—
Cognitive impairment/confusion	11 (26.2)	—	—	—	—
Seizures	8 (19.0)	—	—	—	—
Aphasia	6 (14.3)	—	—	—	—
Cerebellar symptoms/ataxia	5 (11.9)	—	—	—	—
Visual disturbance	4 (9.5)	—	—	—	—
Sensory deficit	4 (9.5)	—	—	—	—
Incidental diagnosis	2 (4.8)	—	—	—	—

Data are presented as mean ± standard deviation (SD) or *n* (%), as appropriate. Continuous variables were compared using the Kruskal–Wallis test, whereas categorical variables were compared using Fisher’s exact test. The three-category brain-metastasis comparison used the Fisher–Freeman–Halton exact test. Abbreviations: GPA, Graded Prognostic Assessment; KPS, Karnofsky Performance Status; EQ-VAS, EuroQol Visual Analogue Scale. † Patients could present with more than one symptom; therefore, percentages do not total 100%. Primary tumor origin and preoperative clinical presentation were reported descriptively and were not included in comparative statistical analyses.

**Table 2:** Association between baseline GPA and postoperative HRQoL improvement and effect sizes for exploratory GPA-category comparisons.

Outcome	Poor	Intermediate	Favorable	P (ε^2^)	Spearman ρ	*p*-Value
ΔEQ-VAS (1 month)	20.0 ± 34.6	32.5 ± 31.4	54.0 ± 18.3	0.035 (0.12)	0.51	<0.001
ΔEQ-VAS (6 months)	26.7 ± 46.2	29.4 ± 27.2	52.3 ± 16.4	0.013 (0.17)	0.50	<0.001
ΔKPS	6.7 ± 15.3	1.3 ± 12.3	2.7 ± 12.2	0.731	0.01	0.933

Data are presented as mean ± standard deviation (SD). Change score (Δ) was calculated as the postoperative score minus the preoperative score. Between-group comparisons were performed using the Kruskal–Wallis test, and correlations with continuous GPA scores were assessed using Spearman’s rank correlation coefficient. Abbreviations: GPA, Graded Prognostic Assessment; HRQoL, health-related quality of life; EQ-VAS, EuroQol Visual Analogue Scale; KPS, Karnofsky Performance Status.

**Table 3:** Longitudinal changes in EQ-5D-5L domain scores across the study follow-up.

EQ-5D-5L Dimension	Preoperative Median (IQR)	1 Month Median (IQR)	6 Months Median (IQR)	Friedman χ^2^	Kendall’s W	Holm-Adj *p*-Value
Mobility	3.0 (2.0–4.0)	1.0 (1.0–2.0)	1.0 (1.0–2.0)	36.60	0.44	<0.001
Self-care	4.0 (3.0–4.0)	2.0 (1.2–3.0)	1.0 (1.0–2.0)	38.11	0.45	<0.001
Usual activities	3.0 (3.0–4.0)	2.0 (1.0–2.8)	2.0 (1.0–2.0)	35.84	0.43	<0.001
Pain/discomfort	4.0 (3.0–4.0)	2.0 (2.0–2.0)	2.0 (1.0–2.0)	49.29	0.59	<0.001
Anxiety/depression	4.0 (3.0–5.0)	2.0 (2.0–2.0)	2.0 (2.0–2.0)	35.79	0.43	<0.001

Values are presented as median (interquartile range [IQR]). Lower EQ-5D-5L scores indicate fewer health-related problems. Longitudinal changes were evaluated using the Friedman test, and Kendall’s W was calculated as the effect-size measure for repeated comparisons. Abbreviation: EQ-5D-5L, EuroQol Five-Dimension Five-Level questionnaire.

## Data Availability

The datasets generated and analyzed during the current study are available from the corresponding author upon reasonable request.

## Supplementary Materials

The following supporting information can be downloaded at https://www.mdpi.com/article/10.3390/jcm15166166/s1, Table S1. Proportion of patients reporting favorable EQ-5D-5L health states during follow-up; Table S2. Preoperative and postoperative Karnofsky Performance Status in the complete study cohort.

## Abbreviations

BM	Brain Metastases
CNS	Central Nervous System
EQ-5D-5L	EuroQol Five-Dimension Five-Level Questionnaire
EQ-VAS	EuroQol Visual Analogue Scale
GPA	Graded Prognostic Assessment
HRQoL	Health-Related Quality of Life
IQR	Interquartile Range
IRB	Institutional Review Board
KPS	Karnofsky Performance Status
PROMs	Patient-Reported Outcome Measures
SD	Standard Deviation
