# Supplementary material for: Baseline Graded Prognostic Assessment Is Associated with Postoperative Health-Related Quality of Life Recovery After Brain Metastasis Resection: A Prospective Cohort Study
Source: J Clin Med. 2026 Aug 8;15(16):6166. doi: 10.3390/jcm15166166 (PMC13513009; doi:10.3390/jcm15166166)
Supplement: Supplementary file 1 [file jcm-15-06166-s001.zip › jcm-4454664-supplementary.pdf]

Supplementary Table S1. Proportion of patients reporting favorable EQ-5D-5L health states during follow-up

EQ-5D-5L dimension	Preoperative, n (%)	1 month, n (%)	6 months, n (%)
**Mobility**	13 (31.0)	36 (85.7)	39 (92.9)
**Self-care**	9 (21.4)	30 (71.4)	41 (97.6)
**Usual activities**	7 (16.7)	31 (73.8)	38 (90.5)
**Pain/discomfort**	6 (14.3)	32 (76.2)	42 (100.0)
**Anxiety/depression**	7 (16.7)	32 (76.2)	32 (76.2)

Favorable health states were defined as EQ-5D-5L response levels 1 or 2, indicating no problems or slight problems. Percentages are based on 42 patients at each assessment.

Supplementary Table S2. Preoperative and postoperative Karnofsky Performance Status in the complete study cohort

KPS assessment	Mean ± SD	Median (IQR)	n
**Preoperative KPS**	77.9 ± 14.7	80.0 (70.0–90.0)	42
**Postoperative KPS**	80.0 ± 11.7	80.0 (70.0–90.0)	42
**Postoperative change in KPS**	2.1 ± 12.2	0.0 (0.0–10.0)	42

Paired preoperative–postoperative KPS comparison: Wilcoxon signed-rank test, W = 153.0, p = 0.372. Postoperative KPS refers to the postoperative assessment recorded in the study dataset. Because the exact postoperative assessment time point was not coded in the spreadsheet, no month-specific interpretation was made.
